# Interleukin-1 receptor (IL-1R) mediates epilepsy-induced sleep disruption

**DOI:** 10.1186/s12868-016-0309-0

**Published:** 2016-11-22

**Authors:** Tzu-Rung Huang, Shuo-Bin Jou, Yu-Ju Chou, Pei-Lu Yi, Chun-Jen Chen, Fang-Chia Chang

**Affiliations:** 1 Department of Veterinary Medicine, School of Veterinary Medicine, National Taiwan University, No. 1, Sec. 4., Roosevelt Road, Taipei, 106 Taiwan; 2 Department of Neurology, Mackay Memorial Hospital and Mackay Medical College, Taipei, Taiwan; 3 Department of Sport Management, College of Tourism, Leisure and Sports, Aletheia University, New Taipei City, Taiwan; 4 Department of Biochemical Science and Technology, College of Life Science, National Taiwan University, Taipei, Taiwan; 5 Graduate Institute of Brain and Mind Sciences, College of Medicine, National Taiwan University, Taipei, Taiwan; 6 Graduate Institute of Acupuncture Science, College of Chinese Medicine, China Medical University, Taichung, Taiwan

**Keywords:** Amygdala, Epilepsy, IL-1 receptor, Kindling, Sleep

## Abstract

**Background:**

Sleep disruptions are common in epilepsy patients. Our previous study demonstrates that homeostatic factors and circadian rhythm may mediate epilepsy-induced sleep disturbances when epilepsy occurs at different zeitgeber hours. The proinflammatory cytokine, interleukin-1 (IL-1), is a somnogenic cytokine and may also be involved in epileptogenesis; however, few studies emphasize the effect of IL-1 in epilepsy-induced sleep disruption. We herein hypothesized that IL-1 receptor type 1 (IL-1R1) mediates the pathogenesis of epilepsy and epilepsy-induced sleep disturbances. We determined the role of IL-1R1 by using IL-1R1 knockout (IL-1R1 −/− KO) mice.

**Results:**

Our results elucidated the decrease of non-rapid eye movement (NREM) sleep during the light period in IL-1R −/− mice and confirmed the somnogenic role of IL-1R1. Rapid electrical amygdala kindling was performed to induce epilepsy at the particular zeitgeber time (ZT) point, ZT13. Our results demonstrated that seizure thresholds induced by kindling stimuli, such as the after-discharge threshold and successful kindling rates, were not altered in IL-1R −/− mice when compared to those obtained from the wildtype mice (IL-1R +/+ mice). This result suggests that IL-1R1 is not involved in kindling-induced epileptogenesis. During sleep, ZT13 kindling stimulation significantly enhanced NREM sleep during the subsequent 6 h (ZT13-18) in wildtype mice, and sleep returned to the baseline the following day. However, the kindling-induced sleep alteration was absent in the IL-1R −/− KO mice.

**Conclusions:**

These results indicate that the IL-1 signal mediates epilepsy-induced sleep disturbance, but dose not participate in kindling-induced epileptogenesis.

## Background

Epilepsy results from the imbalance of excitability and inhibition of neuronal networks [1, 2]. Patients with different types of epilepsy may experience either daytime sleepiness or nighttime sleep disturbance [3–6]. The prevalence of sleep disorders, such as excessive daytime sleepiness [7, 8], insomnia [9, 10] and obstructive sleep apnea [8, 10] is higher among patients with epilepsy.

Interleukin-1β (IL-1β), one of the somnogenic factors, enhances non-rapid eye movement (NREM) sleep by acting at the basal forebrain [11] and the ventrolateral preoptic area (VLPO) [12]. Two types of IL-1 receptors (IL-1Rs) have been identified: the type 1 receptor (IL-1R1) and the type 2 receptor (IL-1R2). IL-1R1 dominantly distributes throughout the brain and carries out the main function of NREM sleep enhancement [13]. IL-1R1 KO mice decrease sleep during the dark period of the light:dark cycle when compared with the wildtype mice [13]. IL-1 is also identified as one of the proinflammatory cytokines, which leads to the pathogenesis of epilepsy during inflammation. The concentrations of IL-1β increase and the number of IL-1 receptors are up-regulated during seizures [14, 15]. Existing evidence indicates that the increased concentrations of IL-1β may affect the seizure threshold, but the role of IL-1β in the epileptogenesis is still controversial, as both a proconvulsant and an anticonvulsant have been suggested. Growing evidence supports IL-1 as a proconvulsant substance. Seizure activities are inhibited after the injection of the IL-1 receptor antagonist (IL-1ra) and seizures are also suppressed in mice with over-expressed IL-1ra [14, 16]. On the other hand, the anticonvulsant effects of IL-1 are also suggested in other studies. IL-1 augments the effect of gamma-aminobutyric acid (GABA)-A receptors and increases the pentylenetetrazol (PTZ)-induced seizure threshold in mice [17]. Intracerebroventricular (ICV) injections of IL-1β suppress the amygdaloid kindling-induced seizures in rats [18]. Furthermore, IL-1 exhibits a temporal and dose-dependent influence on sleep regulation. Low doses of IL-1 increase NREM sleep during the night and slow wave activity during the day; in contrast, high doses of IL-1 suppress NREM sleep during the day and slow wave activity at night [19]. The opposite actions of high- and low-doses of IL-1 may explain the divergent effects. Because of the conflicts between those observations, the role of IL-1 in epileptogenesis needs to be further determined. We herein hypothesized that IL-1R1 mediates the pathogenesis of epilepsy and the epilepsy-induced sleep disturbances. In this study, we examined IL-1R1 in the kindling-induced epileptogenesis and sleep disruptions by employing IL-1R1 −/− mice.

Our previous results have demonstrated that epilepsy occurring at different zeitgeber time (ZT) points alters sleep differently [20, 21]. Kindled epilepsy at ZT0 decreases NREM sleep during the light period; in contrast, kindling stimulation at ZT13 increases NREM sleep during the dark period [20]. At ZT0, corticotropin-releasing hormone (CRH) mediates kindling-induced NREM sleep reduction, and at ZT13, the increases in IL-1 are attributed to kindling-induced NREM sleep enhancement [20]. Furthermore, we further found that at ZT6, kindling epilepsy shifts the fluctuation of period circadian protein homolog 1 protein (PER1) in the suprachiasmatic nucleus (SCN) of the hypothalamus and alters sleep circadian rhythm [20]. These results suggest that epilepsy may alter either homeostatic factors or circadian rhythms to cause sleep disturbances. In this study, we further determined our hypothesis that at ZT13, IL-1R1 mediates kindling-induced sleep disruption by using IL-1R1 +/+ and IL-1R1 −/− mice.

## Methods

### Animals

Male C57BL/6 (IL-1R1 +/+ and IL-1R1 −/−) mice (4- to 6-weeks old) were used in present study. The original IL-1R1 −/− KO mice were obtained from Jackson Laboratory (strain: B6.129S7-*I/1r1* ^*tm1Imx*^/J) and bred in house. The wildtype C57BL/6 mice (IL-1R1 +/+) were purchased from BioLASCO Taiwan Co., Ltd. The polymerase chain reaction (PCR) analysis of brain tissue was performed to confirm the genotype of IL-1R1 KO mice used in experiments and to confirm any genetic drift during breeding (the detail genotyping methods as described later). All experiments and animal care were performed following the principles outlined in the Institutional Animal Care and Use Committee (IACUC) of National Taiwan University. Mice were anesthetized with zoletil (20 mg/kg, i.p.) and xylazine (12 mg/kg, i.p.), treated with antibiotics (penicillin G benzathine) to prevent infection, and surgically implanted with two electroencephalographic (EEG) electrodes (wire-wrapping-wire 30 AWG) and a bipolar stimulating electrode. The placements of EEG electrodes were at the left frontal lobe and right parietal lobe. The bare ends of the insulated leads from the EEG electrodes were connected to Dupont female terminals and a 2.54 mm 2P Dupont connector. Bipolar insulated electrodes (model # M148340, California fine wire company, Grover beach, CA) were placed in the left basolateral nucleus of the amygdala (BLA) as the target of kindling stimuli. The coordinates of the BLA were 1.9 mm caudal to bregma, 2.8 mm lateral to bregma, and 4.6 mm ventral to the dura [22]. The Dupont connector and bipolar electrodes were cemented to the skull with dental acrylic (Tempron, GC Co., Tokyo, Japan). Ibuprofen was added to their drinking water for 5 days after the surgery to reduce pain. Five days after the EEG implantation, the Dupont connector was connected to the amplifier system for habituation. All animals were housed separately in a recording cage and were housed in a 12:12 h light:dark (L:D) cycle in an isolated room where the temperature was maintained at 23 ± 1 °C. Food and water were provided ad libitum.

### PCR genotyping

**Fig. 1:**
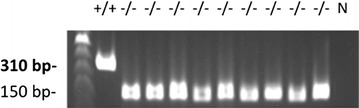
IL-1R1 expression of the cerebral hemispheres from IL-1R +/+ and −/− mice. −/− represents IL-1R1 −/− mice; +/+ refers to wildtype mice. *N* indicates the negative control

For the PCR genotyping analysis in each mouse, 0.5 cm sections of the tail tips were dissolved in 0.2 ml of DirectPCR Lysis Reagent (Viagen Biotech) and 0.5 mg/ml of proteinase K (Roche) solutions under the following conditions: 55 °C for 6–7 h, 85 °C for 45 min, 25 °C for 5 min (1 cycle), and then precipitated by centrifuging for 10 s. One μl of lysate was used for 50 μl PCR reactions with MyTaq HS Mix (Bioline, Taunton, MA). We used the primers recommended by Jackson Laboratory for genotyping (Table 1). The expected PCR band for IL1R1 +/+ is 310 bps and the band for IL-1R1 −/− is 150 bps. The heterozygous (IL-1R+/−) mice revealed both bands of 310 and 150 bps. PCR was performed by a C1000 thermocycler (BioRad, Hercules, CA). The parameters for reaction temperature cycles were 94 °C for 2 min, and then 10 cycles of 94 °C for 20 s, 65 °C for 15 s (−0.5 °C decreases per cycle), and 68 °C for 10 s, followed by 28 cycles of 94 °C for 15 s, 60 °C for 10 s, and 72 °C for 10 s. We analyzed 5 μl of PCR reactions by using 1.5% agarose gel electrophoresis (agarose powders and 0.5× TBE buffer were purchased from Ameresco, Solon). All samples and DNA markers (100 bp DNA ladder, GeneDirex) were mixed with 6× loading buffers (GeneDirex novel juice) and slowly loaded into the slots of submerged gel in the electrophoresis chamber (Mupid-II; Cosmo Bio Co., Tokyo). The gels were run at 100 V for 20 min. We examined the gel by UV illumination and photographed the gel as shown in Fig. 1. The primers are listed in Table 1. Mice used in this experiment were genotyped to confirm no genetic drift (Fig. 1).

**Table 1:** The primer list

Primer	Sequence 5′ to 3′	Primer type
10774	CTCGTGCTTTACGGTATCGC	Mutant forward
20665	GGTGCAACTTCATAGAGAGATGA	Wildtype forward
20666	TTCTGTGCATGCTGGAAAAC	Common

**Fig. 2:**
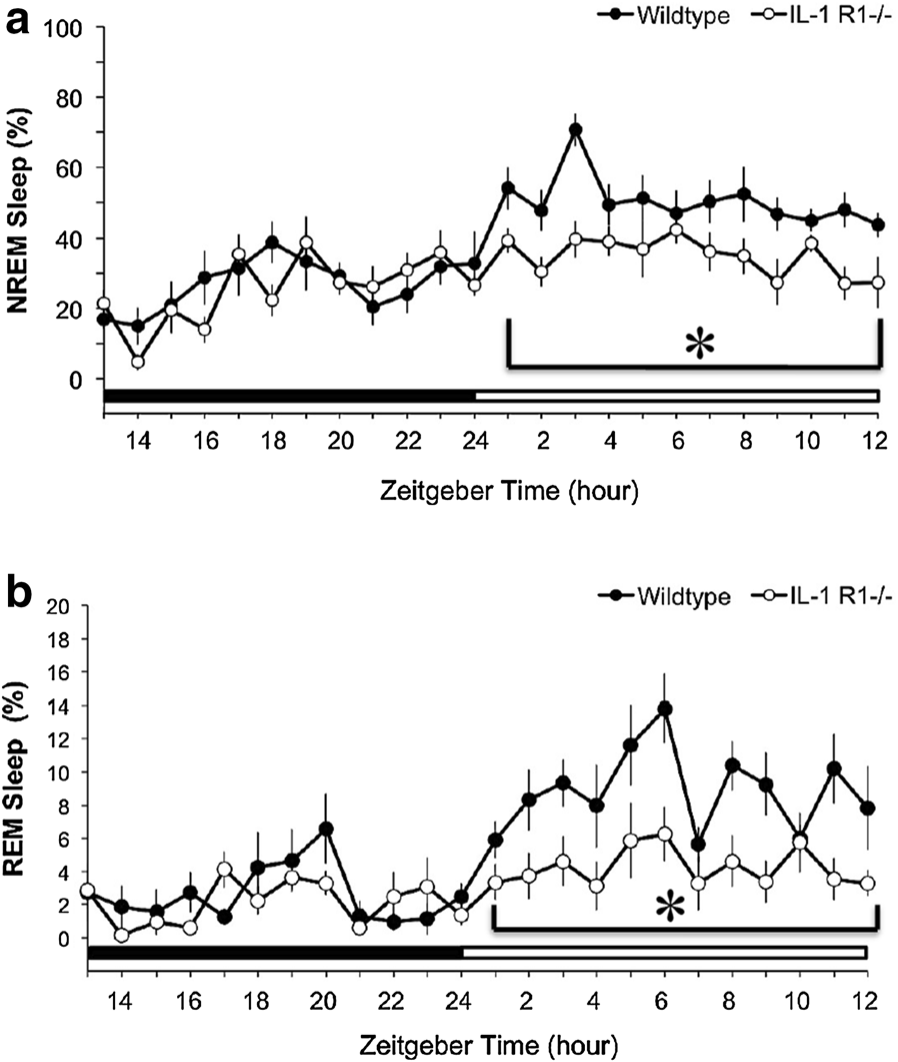
Sleep difference between IL-1R1 +/+ and IL-1R1 −/− mice. **a** Represents the data of NREM sleep and **b** indicates the values of REM sleep. *Closed circles* represent the data obtained from the IL-1R1 +/+ mice, and *open circles* represent the data obtained from the IL-1R1 −/− mice. *Asterisk* refers to a statistically significant difference between two groups. The *black bar* indicates the 12-h dark period and the *white bar* indicates the 12-h light period

### Kindling manipulation

Nine days after surgery, mice were treated with rapid electrical amygdala kindling (REAK) [23, 24] to develop epilepsy. A 2-channel general-purpose stimulus generator (#STG 4002, Multi Channel System MCS GmbH, Reutlingen, Germany) was used to deliver the kindling stimuli. The after-discharge threshold (ADT) was determined for each mouse by giving a series of stimulus intensities, which started from 20 μA and increased by 20 μA every 2 min until epileptiform after-discharge (AD) spikes appeared on EEGs within 10 s after the stimulation. AD spikes are defined as spikes of over 2 Hz and at least three times higher than the baseline EEG amplitude. Mice, not manifesting any AD spikes with a stimulus intensity of 250 μA were excluded from the experiment. The intensity of kindling stimuli depended on individual ADT intensity of each mouse. A total of 40 stimuli were given through the bipolar electrode into the BLA. The stimulus was a train of monophasic pulses (1-ms duration each) of 100 Hz for 3 s, and the stimulation was given every 5 min over a total of 200 min. The severity of seizures was scored according to the 7 grade modifications of Racine’s classification: stage 1, facial clonus; stage 2, head nodding; stage 3, unilateral forelimb clonus; stage 4, rearing with bilateral forelimb clonus; stage 5, rearing and falling (loss of postural control); stage 6, running or bouncing seizures; stage 7, tonic hind limb extension [25, 26]. Mice that exhibited epileptiform EEGs induced by a single stimulus with the intensity of 300 μA at ZT13 after the REAK protocol were considered as a successful induction of epilepsy by kindling.

### Experimental protocols

Two groups of mice were used in this study. Group 1 (n = 13) was used to determine the normal sleep and sleep alteration induced by kindling in wildtype (IL-1R1 +/+) mice at ZT13 and group 2 (n = 18) was used to determine the kindling-induced sleep disturbance in the IL-1R1 −/− mice at ZT13. All recordings started at the beginning of the dark period (ZT13) and continued for 24 h (ZT13-ZT24 and ZT1-ZT12). A 24-h baseline EEG was acquired as the control before the REAK protocol, and no electrical impulses were given at any time before and throughout the baseline recording. Then, 40 stimuli of REAK were given through the bipolar electrode into the left basolateral amygdala (BLA). Mice in all groups received a ZT13-kindling stimulus on the next day (the day of seizure induction) after the REAK procedure. Until epileptiform spikes induced by the ZT13-kindling manifested on EEGs, the mice were continuously recorded for 2 days (1st- and 2nd-day after seizure induction) to evaluate their sleep alteration.

### Statistical analysis for sleep recordings

All values of sleep recordings are presented as mean ± standard error of the mean (SEM). Repeated measures analysis of variance (ANOVA) was performed to analyze the differences in the vigilance states and sleep-architecture parameters across the dark period, light period and specific time blocks. If statistically significant differences were detected, post hoc (Duncan’s) multiple range tests were used. An α level of *p* value <0.05 was taken as indicating a statistically significant difference between groups.

### Statistical analysis for seizure threshold

All ADT intensities are presented as mean ± SEM. Two-way ANOVA was performed to evaluate the differences of ADT intensities between groups, and between successful-kindled and failure-kindled mice. The successful rate of kindling is presented as the percentage. Fisher’s exact test was performed to compare the successful kindling rates between groups. Mann–Whitney *U* test was used to examine the differences of Racine’s stage seizures in successful-kindled mice. The *p* value <0.05 was taken as indicating a statistically significant difference between groups.

## Results

### The role of IL-1R1 in kindling-induced epileptogenesis

The ADT intensities, successful rates of kindling and Racine’s stages of seizure were used to determine the changes in seizure thresholds. Table 1 delineated the results of ADT intensities and the successful rates of kindling. Some mice failed to develop epilepsy, even though they exhibited an AD spike. The ADT intensities between successful-kindled mice and failure-kindled mice were not significantly altered in two groups (*p* = 0.52). The average ADT intensity for success-kindled IL-R1 +/+ mice was 52.7 ± 10.2 μA (n = 11), and was 50 ± 10.0 μA (n = 2) for failure-kindled IL-1R1 +/+ mice. In IL-1R1 −/− mice, the average ADT intensity of success-kindled mice was 69.2 ± 14.8 μA (n = 13) and that of failure-kindled mice was 100 ± 29.7 μA (n = 5). The ADT intensities between IL-1R1 +/+ and IL-1R1 −/− mice were not significantly different (Table 2).

**Table 2:** Results of seizure threshold in two groups of mice

Group	Success kindled		Success kindling rate, %	Failure kindled		Failure kindling rate, %	Total (n)
	(n)	ADT, μA		(n)	ADT, μA		
Wildtype	11	52.7 ± 10.2	84.6	2	50.0 ± 10.0	15.4	13
IL-1R1 −/−	13	69.2 ± 14.8	72.7	5	100.0 ± 29.7	27.3	18

Values of ADT are mean ± SEM. ADT differences were detected by two-way ANOVA

Values of rates are presented as percentage. Fisher’s exact test was performed to compare the difference between two groups of mice

*ADT* after-discharge threshold

The successful rates of developing epilepsy by kindling stimuli in the IL-1R1 +/+ and IL-1R1 −/− were 84.6 (11 out of 13) and 72.2% (13 out of 18), respectively. The successful rates of developing epilepsy in two groups of mice were unremarkable (*p* = 0.73).

The seizures were scored by Racine’s stage scores. No statistically significant difference among the median scores of Racine’s stage between IL-1R1 +/+ and IL-1R1 −/− mice was discovered. The median scores of Racine’s stage in two groups were the same, which were rated as grade 2 (n = 11 for wildtype, n = 13 for IL-1R1 −/−; *p* = 0.6829).

### Sleep difference between IL-1R1 +/+ and −/− mice

During the undisturbed condition, IL-1R1 −/− mice exhibited significantly lower NREM sleep during the light period when compared to IL-1R1 +/+ mice. REM sleep was also significantly lower in the IL-1R1 −/− mice during the light period when compared to IL-1R1 +/+ mice. There was no difference in both NREM and REM sleep during the dark period when comparing between IL-1R1 +/+ and IL-1R1 −/− mice. The time spent in NREM sleep during the 12-h light period was 50.5 ± 1.7% in IL-1R1 +/+ mice and 34.8 ± 1.5% in IL-1R1 −/− mice (*p* < 0.05 vs. IL-1R1 +/+; Fig. 2a). The time spent in REM sleep during the 12-h light period was 8.9 ± 0.6% in IL-1R1 +/+ mice and 4.2 ± 0.4% in IL-1R1 −/− mice (*p* < 0.05 vs. IL-1R1 +/+; Fig. 2b).

### ZT13 kindling-induced sleep alterations in IL-1R1 +/+ mice

**Fig. 3:**
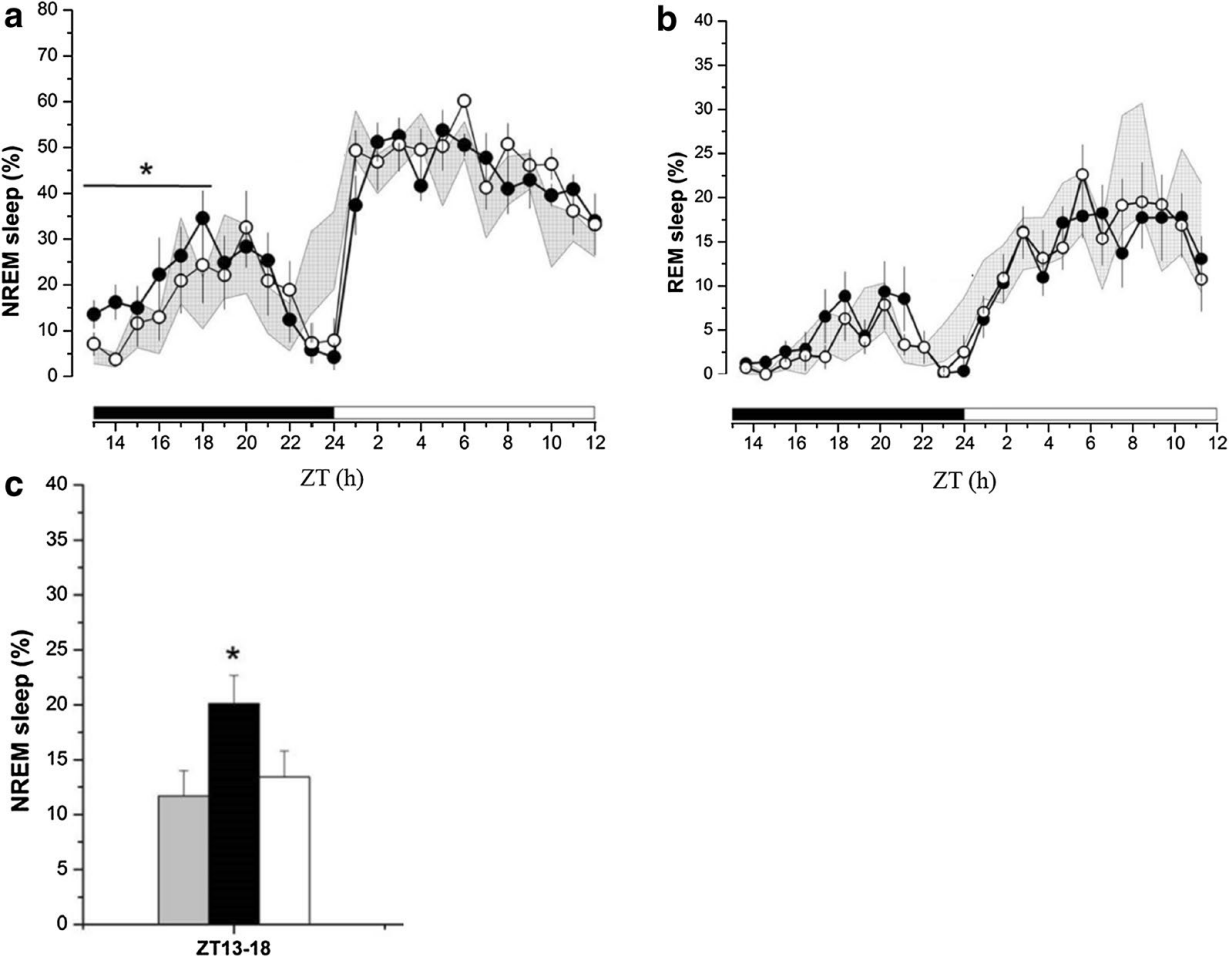
The effects of ZT13 kindling stimuli on sleep alterations in wildtype mice. **a** ZT13 kindling stimuli enhanced NREM sleep during ZT13-18 in the 1st-day after seizure induction, but there was no change on the following day. **b** ZT13 kindling stimulation did not alter REM sleep. *Shadow areas* represents the data obtained from baseline control, *closed circles* represent the data acquired from the 1st-day after seizure induction, and the *open circles* represents the 2nd-day after seizure induction. **c** The summary of NREM sleep alteration after ZT13 kindling stimuli. The *grey bar* represents the data obtained from control, the *black bar* represents the data acquired from the 1st-day after seizure induction, and the *white bar* represents the data obtained from the 2nd-day after seizure induction. *Asterisk* refers to a statistically significant difference between control and the 1st-day after seizure induction. Sleep-wake activity was recorded from the beginning of the dark period (ZT13) and lasted for 24 h

ZT13 kindling stimulation significantly enhanced NREM sleep during ZT13-18 on the 1st-day after seizure induction in IL-1R1 +/+ mice (Fig. 3a). The amount of NREM sleep during ZT13-18 was increased from 11.7 ± 2.3 (obtained before receiving the ZT13 kindling stimulation, the baseline control) to 20.1 ± 2.6% (n = 8) on the 1st-day after seizure induction (*p* < 0.05 vs. baseline control), and returned to 13.5 ± 2.4% on the 2nd-day after seizure induction (*p* > 0.05 vs. baseline control; Fig. 3a, c). The difference of NREM sleep acquired before and after kindling stimulation is 8.6 ± 2.5%. ZT13 kindling stimulation did not alter REM sleep during both dark and light periods on the 1st- and 2nd-day after seizure induction (Fig. 3b).

### ZT13 kindling-induced sleep alterations in IL-1R1 −/− mice

**Fig. 4:**
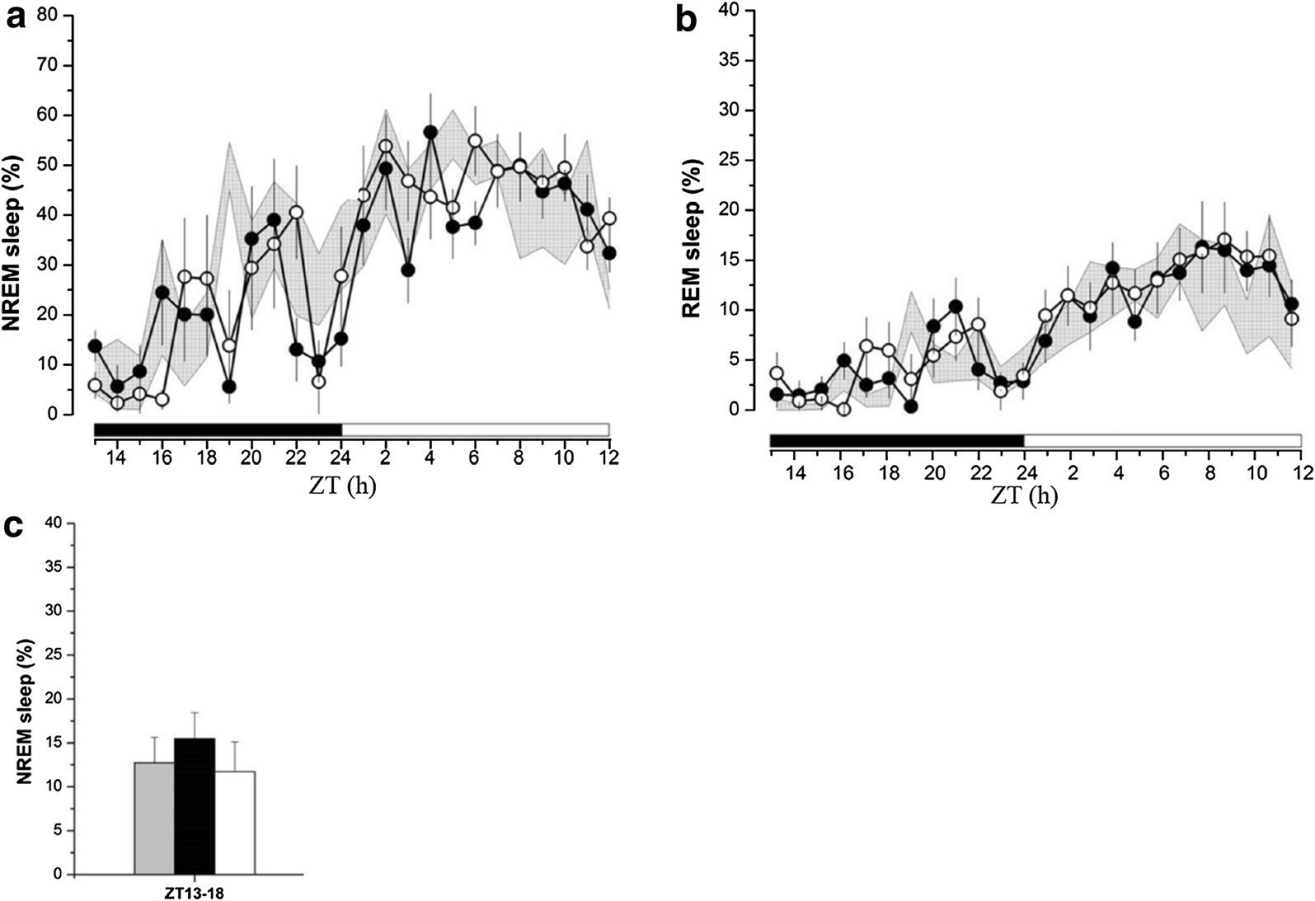
The effects of ZT13 kindling stimuli on sleep alterations in IL-1R1 −/− mice. **a** ZT13 kindling stimuli did not change NREM sleep during ZT13-18 in the 1st- and 2nd-day after successful kindling. **b** ZT13 kindling stimulation did not alter REM sleep either. *Shadow areas* represents the data obtained from baseline control, *closed circles* represent the data acquired from the 1st-day after seizure induction, and the *open circles* represents the 2nd-day after seizure induction. **c** The summary of NREM sleep alteration after ZT13 kindling stimuli. The *grey bar* represents the data obtained from control, the *black bar* represents the data acquired from the 1st-day after seizure induction, and the *white bar* represents the data obtained from the 2nd-day after seizure induction. Sleep-wake activity was recorded from the beginning of the dark period (ZT13) and lasted for 24 h

In IL-1R1 −/− mice, ZT13 kindling stimulation did not have significant impact on NREM sleep during ZT13-18 (Fig. 4a). The time spent in NREM sleep during ZT13-18 was 12.7 ± 2.9% before receiving the ZT13 kindling stimulation (the baseline control), 15.5 ± 3.9% (*p* > 0.05 vs. baseline control) from the 1st-day after seizure induction, and 11.7 ± 3.4% (*p* > 0.05 vs. baseline control) from the 2nd-day after seizure induction (Fig. 4a, c). The difference between NREM sleep acquired before and after kindling stimulation is 2.5 ± 2.6% (*p* < 0.05 vs. the difference obtained from IL-1R1 +/+ mice). Effects of ZT13 kindling stimulation on REM sleep and wakefulness did not reach statistical significance during any of the time blocks (Fig. 4b). The percentages of REM sleep during ZT13-18 obtained from the baseline, the 1st-day, and the 2nd-day after seizure induction were 1.2 ± 0.4, 2.6 ± 0.6 (*p* > 0.05 vs. baseline control), and 3.0 ± 0.9% (*p* > 0.05 vs. baseline control), respectively.

## Discussion

Many epileptic patients suffer from sleep disturbances, which enormously reduce their quality of life [8, 9, 27]. Although the comorbidity of epilepsy and sleep disorders is generally evidenced, the underlying mechanisms have yet to be clarified. This study investigated the roles of IL-1R1 in the thresholds of kindling-induced epileptogenesis and in the kindling-induced sleep disturbances. An IL-1R1 KO mouse was the ideal model to explore the role of IL-1; the difference in parameters can be examined when there is a lack of IL-1 signaling.

The theory assumed to interpret the discrepant role of IL-1 in epileptogenesis derives from the variability of IL-1 concentrations and the complexity of IL-1 downstream cytokines. Therefore, the transgenic IL-1R1 KO mouse is an excellent model to examine the significance of IL-1 when its signal in the brain is completely eliminated. Much evidence also supports the proconvulsant role of IL-1 in epilepsy. In rat models, systemic administration of IL-1ra reduces seizures induced by hippocampal kindling or pilocarpine [14]. In transgenic mice, bicuculline methiodine-induced seizure decreases in IL-1ra over-expressed mice, and the seizure threshold in heating-induced febrile seizure is increased in IL-1R1 −/− mice [16, 28]. IL-1 may enhance seizures by facilitating LTP in the hippocampus; however, it is also suggested that as the dosage is increased, IL-1 performs the opposite role and suppresses LTP [29, 30]. A recently published paper further describes that the pathogenesis and maintenance of temporal lobe epilepsies are attributed to the activation of P2X7 receptors by the release of ATP during neurodegeneration, which activates the microglia to release IL-1β [31]. In contrast, the anticonvulsant role of IL-1β has also been suggested in BLA-kindling rats, as evidenced by the decrease in seizures after ICV injection of IL-1β [18]. Our current results indicate no change in the seizure thresholds in kindling epileptogenesis in mice with a lack of IL-1R1. These results suggest that IL-1R1 is not a critical factor in influencing kindling-induced epileptogenesis. However, our other study demonstrated that IL-1R1 contributes to the epileptogenesis when epilepsy is induced by pentylenetetrazol (PTZ) (personal unpublished result).

IL-1β is one of the potent somnogenic factors. It’s been reported that NREM sleep during the dark period is reduced in IL-1R1 KO mice when comparied to the wildtype mice [13]. In our results, we found that both NREM and REM sleep were reduced during the light period, rather than during the dark period. IL-1β exhibits circadian fluctuation with higher concentrations during the light period and with lower concentrations during the dark period [32]. A lack of IL-1 signaling should affect sleep during the light period, and our results provided the evidence to support the somnogenesis of IL-1 signaling. The increase of NREM sleep after ZT13 kindling stimulus in the wildtype mice, but not in the IL-1R1 KO mice revealing the importance of IL-1 receptors in epilepsy-induced sleep alterations. During the first 6 h following ZT13 kindling stimulus (ZT13-18), NREM sleep obtained from the wildtype mice increased. These alterations returned to the baseline value on the 2nd day. In transgenic knockout mice, kindling stimulation did not alter NREM sleep during first 6 h.

During neuronal damage, the expression of IL-1β and its receptors are increased, and IL-1β signaling pathways are activated when encountering pathological damage [15, 33]. The activation of IL-1β function may facilitate neuronal excitability by modulating long-term potentiation (LTP) and inhibitory GABA receptors in hippocampal areas [17, 30]. Besides affecting synaptic plasticity, the increase of IL-1β influences both NREM sleep and REM sleep. Administration of IL-1β into the CNS, or peripheral circulated system, significantly enhances NREM sleep [34]. But when the dose of IL-1β reaches a pathogenic concentration, the opposite result occurs in which both NREM sleep and REM sleep are suppressed [35, 36]. The common phenomenon that NREM sleep increases and REM sleep is suppressed during inflammation in individual is in accordance with the results of IL-1β administration [37]. This conjecture explains the close link between sleep and the immune system, and it helps interpret our results that NREM sleep increased after ZT13 kindling in wildtype mice, but not IL-1R1 KO mice.

The increases of sleep after seizures have also been reported in pilocarpine-induced epileptic rats. Administration of pilocarpine enhances and fragments slow wave sleep in the following night and morning, and returns to normal after 24 h [38]. Quigg et al. [39] demonstrated that deep NREM sleep increases following one kindling stimulus, and lasts even longer after 5 times of accumulated kindling stimuli. In our previous study, the seizure occurrence time is considered as a critical factor in determining the ways to alter sleep; kindling stimulation at ZT0 decreases NREM sleep, whereas ZT13 kindling stimulation enhances NREM sleep [18, 20]. IL-1 mRNA expression increases in the hippocampus and cortex after ZT13 kindling stimulus and the increase of NREM sleep is blocked by administration of IL-1ra [20, 21]. Nevertheless, our current study demonstrated that a lack of IL-1 signals in the IL-1R1 −/− mice failed to express the ZT13-induced sleep enhancement. Furthermore, different IL-1 signals may lead to different outcomes between epilepsy and sleep. For example, IL-1 activates both p38 mitogen-activated protein kinase (MAPK) and nuclear factor κB (NF-κB) pathways in astrocytes, but only regulates p38 MAPK pathways in the hippocampal neurons [40]. Particularly, a lower dose of IL-1 phosphorylates Src kinase in the hippocampal neurons [40]. Furthermore, different isoforms of the IL-1 receptor accessory proteins also mediate different signal pathways [40]; however, the mechanisms need to be further determined. Astrocytes and neurons contribute to both epileptogenesis and sleep regulation. Different IL-1 signaling outcomes could explain why IL-1R1 plays a role in sleep alteration, but not in epileptogenesis in kindled mice.

## Conclusions

In summary, our results demonstrated the essential role of IL-1R1 in kindling-induced sleep disturbance by using transgenic IL-1R1 KO mice. Epilepsy-induced sleep disturbances were absent in the IL-1R1 KO mice, indicating the importance of IL-1 signals. The knockout of IL-1R1 did not change the seizure thresholds, suggesting that IL-1R1 signaling is not in involved in the kindling-induced epileptogenesis.

## Abbreviations

AD: after-discharge
ADT: after-discharge threshold
BLA: basolateral nucleus of amydgala
CRH: corticotropin-releasing hormone
EEG: electroencephalography
GABA: gamma-aminobutyric acid
ICV: intracerebroventricular
IL-1: interleukin-1
IL-1R1: IL-1 receptor type 1
IL-1ra: IL-1 receptor antagonist
KO: knockout
LTP: long-term potentiation
NREM: non-rapid eye movement
PCR: polymerase chain reaction
PER1: period circadian protein homolog 1 protein
PTZ: pentylenetetrazol
REAK: rapid electrical amygdala kindling
REM: rapid eye movement
SCN: suprachiasmatic nucleus
VLPO: ventrolateral preoptic area
ZT: zeitgeber time
